# New colorimetric screening assays for the directed evolution of fungal laccases to improve the conversion of plant biomass

**DOI:** 10.1186/1472-6750-13-90

**Published:** 2013-10-26

**Authors:** Isabel Pardo, Xiomara Chanagá, Ana Isabel Vicente, Miguel Alcalde, Susana Camarero

**Affiliations:** 1 Centro de Investigaciones Biológicas, CSIC, Ramiro de Maeztu 9, 28040 Madrid, Spain; 2 Department of Biocatalysis, Institute of Catalysis, CSIC, Marie Curie 2, 28049 Madrid, Spain

**Keywords:** High-throughput screening, Laccase, Lignocellulose, S-type phenolic mediators, Dyes, Violuric acid

## Abstract

**Background:**

Fungal laccases are multicopper oxidases with huge applicability in different sectors. Here, we describe the development of a set of high-throughput colorimetric assays for screening laccase libraries in directed evolution studies.

**Results:**

Firstly, we designed three colorimetric assays based on the oxidation of sinapic acid, acetosyringone and syringaldehyde with λ_max_ of 512, 520 and 370 nm, respectively. These syringyl-type phenolic compounds are released during the degradation of lignocellulose and can act as laccase redox mediators. The oxidation of the three compounds by low and high-redox potential laccases evolved in *Saccharomyces cerevisiae* produced quantifiable and linear responses, with detection limits around 1 mU/mL and CV values below 16%. The phenolic substrates were also suitable for pre-screening mutant libraries on solid phase format. Intense colored-halos were developed around the yeast colonies secreting laccase. Furthermore, the oxidation of violuric acid to its iminoxyl radical (λ_max_ of 515 nm and CV below 15%) was devised as reporter assay for laccase redox potential during the screening of mutant libraries from high-redox potential laccases. Finally, we developed three dye-decolorizing assays based on the enzymatic oxidation of Methyl Orange (470 nm), Evans Blue (605 nm) and Remazol Brilliant Blue (640 nm) giving up to 40% decolorization yields and CV values below 18%. The assays were reliable for direct measurement of laccase activity or to indirectly explore the oxidation of mediators that do not render colored products (but promote dye decolorization). Every single assay reported in this work was tested by exploring mutant libraries created by error prone PCR of fungal laccases secreted by yeast.

**Conclusions:**

The high-throughput screening methods reported in this work could be useful for engineering laccases for different purposes. The assays based on the oxidation of syringyl-compounds might be valuable tools for tailoring laccases precisely enhanced to aid biomass conversion processes. The violuric assay might be useful to preserve the redox potential of laccase whilst evolving towards new functions. The dye-decolorizing assays are useful for engineering *ad hoc* laccases for detoxification of textile wastewaters, or as indirect assays to explore laccase activity on other natural mediators.

## Background

Laccases catalyze the oxidation of a variety of substituted phenols and many other aromatic compounds without any other requirement than oxygen from air. As a result, these multicopper oxidases are promising green biocatalysts for several industrial sectors such as textile, food, wood and pulp, bioremediation, organic synthesis or electrocatalysis [1-3]. Fungal laccases and, in particular, the high-redox potential laccases (HRPLs) secreted by white-rot basidiomycetes, exhibit high biotechnological applicability due to the wider range of reducing substrates that can be oxidized at the T1 copper site (E_0_ ≈ + 0.8 V, [4]).

Lignin biodegradation is an oxidative process carried out by white-rot fungi in which the breakdown of aryl-ether (β-O-4) linkages and the oxidative degradation of the side chains from the *p*-hydroxyphenyl (H), guaiacyl (G) and syringyl (S) lignin units, releases a set of phenolic compounds (acids, ketones and aldehydes) [5]. Some of them (e.g. acetosyringone, syringaldehyde, *p*-hydroxycinnamic acids) show remarkable activity as laccase redox mediators. Once oxidized by fungal laccases, they act as diffusible electron shuttles, promoting the oxidation of the own lignin polymer and a variety of different recalcitrant aromatic pollutants [6-9]. In recent findings we have highlighted the biotechnological potential of fungal laccases and their natural redox mediators for improving the conversion of plant biomass in the modern integrated lignocellulose biorefinaries [10]. Moreover, sinapic acid, acetosyringone and other bioactive compounds with anti-bacterial and antioxidant properties [11,12] can be used to add new properties to cellulose or wood fibers by grafting reactions catalyzed by laccase [13]. Other phenolic compounds that can be extracted from wood lignin like syringaldehyde or vanillin provide flavor and fragrance or are used as chemical precursors for pharmaceuticals [14,15]. Indeed, the occurrence of lignin-derived phenolic compounds has been profusely described during the processing of lignocellulosic materials. Black liquors from pulp cooking constitute low-cost sources of natural mediators which can be applied in laccase-mediator systems for Totally-Chlorine-Free bleaching of paper pulps [16]. Lignin-related phenolics are also released during the thermo-chemical pretreatment of lignocellulose for bioethanol production, inhibiting the subsequent fermentation step [17]. Detoxification of these slurries can be achieved by the polymerization of these phenols catalyzed by laccase [18], although some of them (vanillin) are somehow resistant [19]. Engineering robust laccases with improved activities/specificities towards the above-mentioned compounds represents a valuable step forward to implement these enzymes in white –industrial– biotechnology processes for conversion of lignocellulosic biomass into chemicals, materials and biofuels [10].

In this scenario, directed molecular evolution constitutes a powerful strategy to adjust the stability and catalytic efficiency of the enzyme to the restrictive industrial operational conditions. On the other hand, it is well known that the availability of high-throughput screening (HTS) assays is mandatory for exploring the enzyme libraries created by random mutagenesis and recombination of the parent gene(s) [20]. Indeed, one of the main bottlenecks in directed evolution originates from the lack of reliable HTS assays specific for the targeted enzyme, and laccase is not an exception. Another major difficulty for engineering fungal laccases, in particular those from basidiomycete fungi, is their tricky heterologous expression. Nevertheless, we have recently reported the successful functional expression of two HRPLs (from *Pycnoporus cinanbarinus* and PM1 basidiomycetes) in *S. cerevisiae* by directed evolution [16,21]. We have also obtained a set of chimeric HRPLs, secreted by yeast, with improved thermostability, diverse pH activity profiles and high-rate oxidation activity as generalist biocatalysts [22-24]. These platforms are good starting points to face up to new challenges such as the design of laccases with improved efficiency towards substrates of biotechnological interest and stable under specific industrial conditions. Promising laccase engineering targets would be the first-order oxidation rate of certain phenolic compounds derived from lignocellulose, to contribute to the integral conversion of plant biomass, or of synthetic organic dyes, for enzymatic removal of color from textile effluents. The development of new HTS assays based on the oxidation of phenolic compounds and organic dyes (under preferred pH and temperature conditions) is of high relevance for the aforementioned purposes.

The current work describes the design and validation of an array of novel HTS assays based on natural compounds derived from lignocellulose and synthetic organic dyes to explore mutant libraries of fungal laccases. Specifically, we developed colorimetric assays based on the oxidation of phenolic compounds related to the S lignin units. These compounds, which are natural substrates of laccases (and ligninolytic peroxidases [25]), might constitute a key step in the enzymatic deconstruction of lignocellulose due to their role as linkages between carbohydrates and lignin in the secondary cell wall of grasses [26]; or they may act as efficient laccase redox mediators promoting the removal of pollutants or complex polymers [16,27,28]. In addition, the oxidation of the artificial mediator violuric acid was devised as reporter assay for the preservation of the redox potential of HRPLs through the evolution procedure. Finally, we performed the development of HTS assays based on the enzymatic oxidation of synthetic dyes either directly or indirectly (in the presence of mediators).

## Results and discussion

### Oxidation of natural phenolic compounds of biotechnological interest

**Figure 1:**
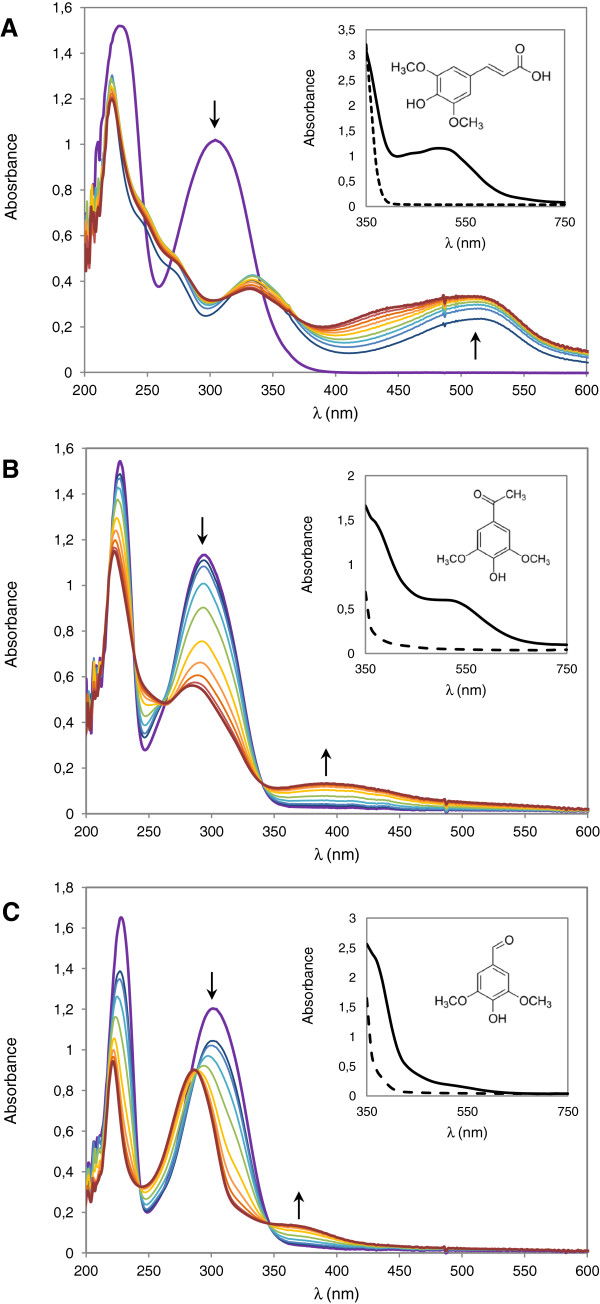
**Oxidation spectra of S-type phenolic compounds.** Changes in the absorption UV-Vis spectra of sinapic acid **(A)**, acetosyringone **(B)** and syringaldehyde **(C)** during oxidation by *M. thermophila* laccase (50 mU) at 0, 1, 2, 5, and 10–60 min (in 10 min intervals) reaction times. Insets show the visible initial spectra (dashed lines) and after 10h (continuous lines).

Among lignin-related phenolic compounds, we chose three S-type phenolic compounds whose enzymatic oxidation generates colored products (acetosyringone, sinapic acid and syringaldehyde) to develop the HTS assays. S-type compounds are easily oxidized by both high- and low-redox potential laccases (LRPLs), as we confirmed here by using the commercial HRPL from *Trametes villosa* (TvL) and the LRPL from *Myceliophthora thermophila* (MtL). The changes in the UV-visible spectra of sinapic acid, acetosyringone and syringaldehyde during their oxidation by laccase showed similar patterns: a rapid decrease of maximum absorbance at 300 nm along with the appearance of absorbance peaks in the visible spectrum (Figure 1). In the case of sinapic acid, we detected a rapid pinkish response (with maximum absorbance around 515 nm) resulting from oxidized dimeric products derived from the dehydrosinapic acid dilactone [29]. Once sinapic acid is oxidized by laccase, the high tendency of its phenoxyl radicals for β-β' coupling are responsible for the accumulation of phenolic dimeric products, which are again oxidized by the enzyme. The oxidation of acetosyringone and syringaldehyde generated an immediate increase of absorbance around 370 nm (yellow color). The color kept stable for syringaldehyde but turned to red in the case of acetosyringone, whose maximum wavelength shifted to 520 nm and was maintained for several hours. Syringaldehyde oxidation finally rendered a strong absorption maximum at 284 nm with a smaller peak at 370 nm, in concordance with the yellow product 2,6-dimethoxy *p*-benzoquinone. The latter has been reported as end product from the enzymatic oxidation of syringaldehyde, acetosyringone, syringic acid or sinapic acid, depending on the reaction conditions [21,29,30].

**Figure 2:**
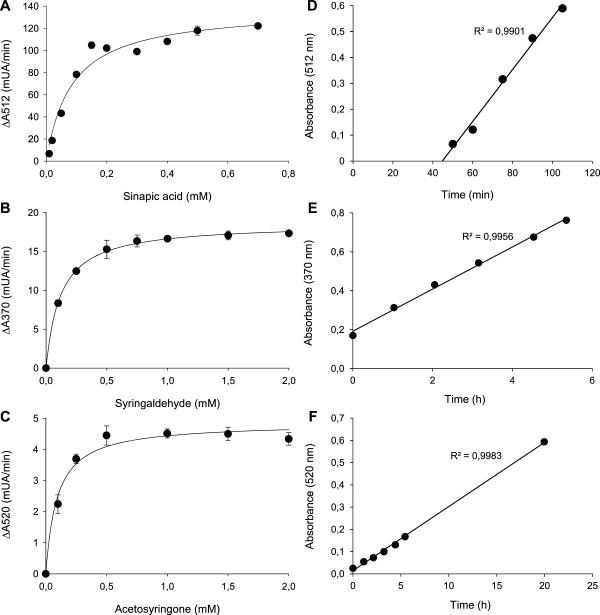
**Determination of conditions for the HTS assays with S-phenolic compounds.** Oxidation rates of TvL (10 mU) for sinapic acid **(A)**, syringaldehyde **(B)** and acetosyringone **(C)** measured at 512 nm, 370 and 520 nm, respectively; and color responses of the endopoint HTS assays with time using 250 μM of sinapic acid **(D)** and 2 mM of syringaldehyde **(E)** and acetosyringone **(F)** with 15 μL of crude extracts from *S. cerevisiae* micro-cultures secreting 3A4 HRPL. Each point represents the mean and SD derived from three independent experiments.

**Figure 3:**
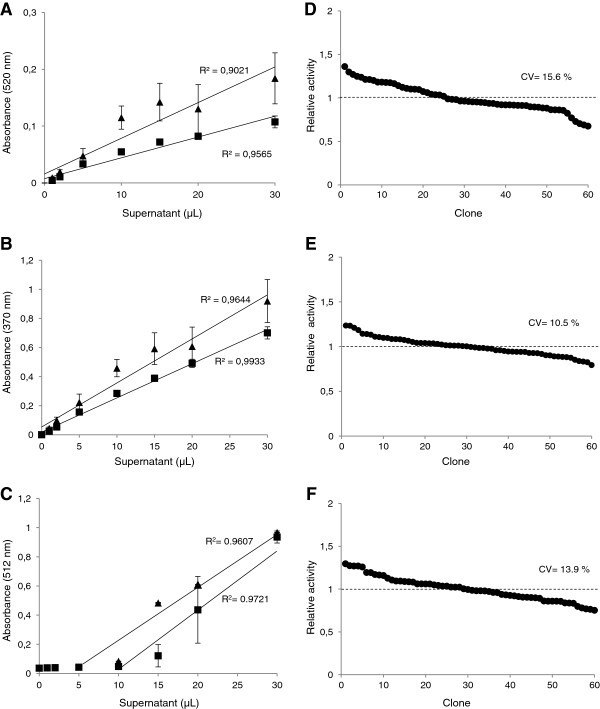
**Validation of HTS colorimetric assays with S-phenolic compounds.** Linearity of the assays based on the oxidation of acetosyringone **(A)** and syringaldehyde **(B)** after 5 h, and sinapic acid **(C)** after 2 h, using crude extracts from *S. cerevisiae* micro-cultures secreting 3A4 HRPL (triangles) or R2 LRPL (squares). Coefficient of variation (CV) of the HTS colorimetric assays based on the oxidation of acetosyringone **(D)** and syringaldehyde **(E)** and sinapic acid **(F)**. Laccase activities from different replicates of the same clone are plotted in descending order.

The λ_max_ for measuring the oxidation of the S-type substrates were established as follows: 512 nm for the sinapic acid's pinkish product, 370 nm for the syringaldehyde's yellow product and 520 nm for the acetosyringone's reddish product. Laccase oxidation rates showed the typical Michaelis-Menten kinetics for the three compounds with K_m_ values of 85, 120 and 93 μM, respectively, for TvL (Figure 2A-C). The concentrations used in the HTS assays (providing stable response without precipitation) were 2 mM acetosyringone or syringaldehyde and 250 μM sinapic acid. The assays were validated using fresh supernatants from the micro-fermentations of *S. cerevisiae* transformed cells secreting laccase (in 96-well plate format). In particular, to check the reproducibility and linearity of the assays, we used *S. cerevisiae* cells expressing either a LRPL, R2 (obtained from the directed evolution of MtL [31]), or a HRPL, 3A4 (a chimeric laccase engineered by family shuffling of evolved PcL and PM1L [24]). The colored responses were feasibly quantified by the increment of absorbance with time (Figure 2D-F), although in the case of the sinapic acid assay an initial lag time was observed due to the multiple oxidation, coupling and cyclization steps required to provide the colored product (oxidized dimer) [29]. Regardless of the compound used, the colored responses were linear (absorbance increased with increasing volumes of supernatant) with both laccases, the LRPL R2 and the HRPL 3A4, expressed by *S. cerevisiae* cells (Figure 3A-C). The lowest detection limits for the acetosyringone and syringaldehyde endpoint assays (carried out in 5 h) were around 0.6 laccase mU/mL (0.15 mU in the well, referred to ABTS activity), whereas, due to the initial lag phase of the sinapic acid assay, 1 mU/mL (0.25 mU in the well) was the lowest activity detected during the 1-2 h of reaction. However, it is worth noting that for longer reaction times, lower laccase activities may also be detected with sinapic acid. The validation of the assays was completed by replicating the same clone in a test 96-well plate and measuring the laccase activities of each well with the target substrate. In all cases, the CV values ranged from 11 to 16% (Figure 3D-F), which is satisfactory to guarantee the reliability of the assays for directed evolution studies.

**Figure 4:**
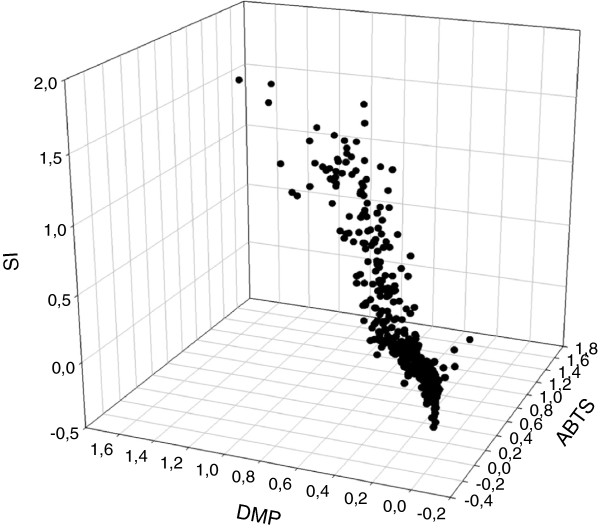
**3D Landscape from the multi-screening with sinapic acid, ABTS and DMP of the laccase mutant library obtained by random mutagenesis and *in vivo *shuffling of HRPLs.** Laccase activities of the clones are depicted in descending order respecting the activity of the best parental (3A4 laccase). Sinapic acid (SI), 2,6-dimethoxyphenol (DMP).

**Figure 5:**
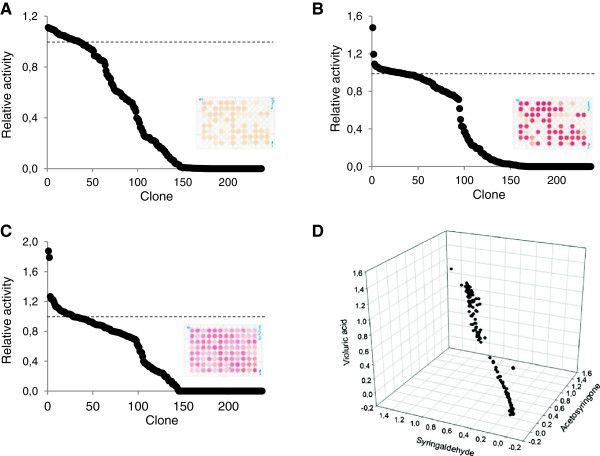
**Multi-screening with mediator compounds.** Landscapes from the HTS of the mutagenic library obtained from 3A4 HRPL using 2 mM syringaldehyde **(A)**, 2 mM acetosyringone **(B)** and 20 mM violuric acid **(C)**. Laccase activities of the clones are depicted in descending order respecting the activity of the parent type. Direct correlation of the activities of the clones explored by the triple screening is shown in the 3D landscape **(D)**.

Finally, the assays were tested for screening mutant libraries of HRPLs secreted by yeast. It is important to highlight that the sinapic acid assay has been recently used to screen mutant libraries generated during the directed evolution of *P. cinnabarinus* laccase (PcL) [22]. In the present study, we used this assay (together with ABTS and DMP assays) to screen a laccase library obtained by random mutagenesis and *in vivo* DNA shuffling of chimeric HRPLs recently engineered in our lab [24]. The 3D landscape obtained from the multi-screening of this library demonstrated that most of the 2000 clones kept the characteristic substrate promiscuity of laccases and some of them showed slight activity improvements respecting the parent types (Figure 4). To complete the study, small libraries of around 250 clones were constructed by error-prone PCR of 3A4 HRPL (with a mutational rate of 1–3 amino acid substitutions per protein sequence) and explored with acetosyringone and syringaldehyde. Landscapes from the dual screening were similar and the data were quite consistent for the two assayed protocols. Approximately 100 clones were inactivated by the mutagenesis and no notable activity increases respecting the parent type were observed (Figure 5A-B). The small size of the mutagenic library probably precludes the selection of remarkable mutants (typically, a directed evolution generation comprises the screening of 2000–3000 clones). Even so, as we could detect slight differences in laccase activity among the different clones, the sensitivity of the colorimetric assays was confirmed.

**Figure 6:**
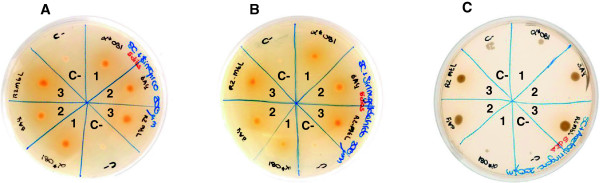
**Screening in solid format.***Saccharomyces cerevisiae* cells secreting different laccase mutants grown for 5 days in SC plates supplemented with sinapic acid **(A)**, syringaldehyde **(B)** or acetosyringone **(C)**. The colored halo around the colonies demonstrated the secretion of laccase (1 and 2 correspond to HRPL mutants, and 3 to LRPL mutant) as compared with the negative control (C-).

It is worth mentioning that the abovementioned S-phenolic substrates might also be used for pre-screening of mutant laccase libraries in solid format. We cultured fresh *S. cerevisiae* laccase transformants on agar-SC-expression plates supplemented with acetosyringone, syringaldehyde or sinapic acid. Laccase secretion was detected by the presence of intense colored halos around the colonies grown in plates supplemented with syringaldehyde or sinapic acid, as compared with the negative control. The intensity of the halos when using acetosyringone was, however, much less intense (Figure 6).

Lignin-related phenolics have been proved to mediate the *in vitro* degradation of recalcitrant aromatics by laccase [6,8], and they constitute an alternative for expensive artificial mediators such as 1-hydroxybenzotriazol (HBT) or violuric acid. Moreover, by contrast to the restricted use of HBT or violuric acid as mediators of HRPLs [32], S-type phenolic mediators from lignocellulosic feedstock could be applied with other laccases. Indeed, S-phenolic compounds notably promote oxidative reactions catalyzed by LRPLs such as MtL from the ascomycete *M. thermophila*[33,34] or even by bacterial laccases with lower redox potentials [21]. This fact is of high interest for the biotechnological application of ascomycete or bacterial laccases that have the advantage of being more easily amendable by protein engineering than basidiomycete laccases. The prompt oxidation of the S-type phenolic compounds (due to the presence of two methoxyl substituents in the aromatic ring) make feasible the use of the new HTS assays for directed evolution studies of LRPLs or other phenoloxidases depicted in bacterial genome databases [35].

### Violuric acid as reporter assay for assessing redox potentials during protein engineering

**Figure 7:**
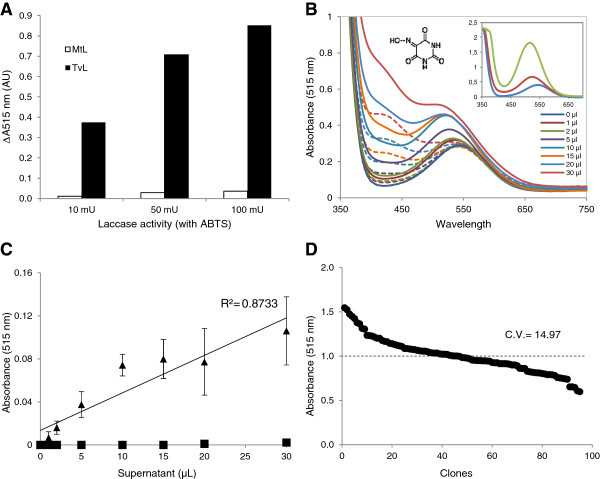
**Validation of violuric acid HTS assay.** Oxidation of 20 mM violuric acid (VIO) by MtL (white bars) and TvL (black bars) using equal laccase activity units respecting ABTS **(A)**. Changes in the visible spectrum of 20 mM VIO after oxidation (t0, dashed lines; 20 h, continuous lines) by different volumes of crude cell extracts from *S. cerevisiae* micro-cultures secreting 3A4 HRPL (the inset shows the oxidation of VIO by 100 mU TvL in 0, 1 and 20 h) **(B)**. Linearity of the HTS assay using crude extracts from *S. cerevisiae* micro-cultures secreting 3A4 HRPL (triangles) or R2 LRPL (squares) **(C)**. Reproducibility of the endpoint HTS assay based on the oxidation of 20 mM VIO using crude cell extracts from replicates of the same clone expressing 3A4 HRPL (20 μL supernatant). The activities are plotted against the clones in descending order **(D)**.

Unlike the straightforward oxidation of S-type phenolic compounds, violuric acid is effectively oxidized only by HRPLs due to its high-redox potential (E_0_ ≈ +1.1 V) [32]. The distinct oxidation rates of violuric acid by low (MtL) and high-redox potential (TvL) fungal laccases confirmed this assessment (Figure 7A). When engineering fungal laccases a single amino acid change in the coordinating sphere of T1 copper may alter the complex modulation of laccase redox potential [36]. Thus, it would be helpful to have an assay to check if the high redox potential of the parental type is being maintained in the selected mutants. We devise here a HTS colorimetric assay based on the oxidation of violuric acid as an easy method to initially evaluate the redox potential of the laccase mutants generated through directed evolution of fungal laccases.

The oxidation of violuric acid generates very stable iminoxyl radicals (without dimerization products) whose purple color can be detected and quantified in the visible spectrum (λ_max_ around 515 nm) [37]. The color turned to reddish when using crude extracts from *S. cerevisiae* micro-cultures due to coupling of violuric radicals to the Cu^2+^ ions from the expression medium, which produces an increment of absorbance at 420 nm [38]. Nevertheless, the increment of absorbance at 515 nm could be measured without interferences (Figure 7B). Crude extracts of *S. cerevisiae* cells secreting laccase in microplate wells were used to validate the assay. We used 20 mM violuric acid because, though it was not a saturating concentration, it rendered soluble and quantifiable colored response with absorbance values within the plate reader's detection limit. With a CV around 15% and high linearity, the assay worked for the evolved HRPL (3A4), whereas, as expected, no oxidation of violuric acid was obtained with the evolved LRPL (R2), even when both crude cell extracts showed closely similar activity on ABTS (around 120 mU/mL supernatant) (Figure 7C-D). The lowest detection limit for this assay was around 0.6 mU/mL supernatant (0.15 mU in the well).

Finally, the mutagenic library obtained by error-prone PCR from the evolved HRPL 3A4 was also screened with violuric acid as substrate (Figure 5C). We observed a direct correlation among the activities of the clones with violuric acid, acetosyringone and syringaldehyde (Figure 5D). In other words, active mutants on S-type phenolic compounds were also capable of oxidizing violuric acid. Thus, by using this reporter assay, we can assess whether the high redox potential of a parental laccase is preserved in all the active mutants generated through the evolution pathway or not.

### Decolorization of synthetic organic dyes

**Figure 8:**
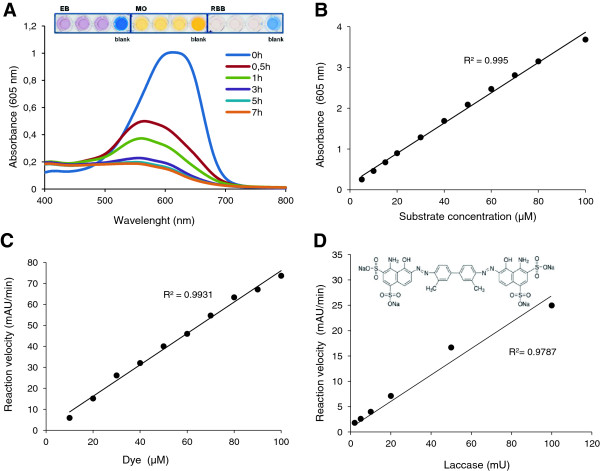
**Oxidation of Evans Blue by laccase.** Changes in the absorbance visible spectrum of Evans Blue dye (EB) during its oxidation by 20 mU *T. villosa* laccase (TvL). The inset shows the colorimetric (decolorizing) response obtained using 50 μM EB, 50 μM Methyl Orange (MO) and 200 μM Remazol Brilliant Blue (RBB) after 30 min of reaction with 10 mU TvL in triplicate **(A)**. Correlation between concentration of EB and maximum peak absorbance **(B)**. Oxidation velocities of EB by TvL (200 mU) measured by the decrease of absorbance at 605 nm using dye concentrations within the plate reader’s detection limit **(C)**. Decolorization of EB (50 μM) as absorbance decrease/min using different amounts of TvL **(D)**.

Three synthetic organic dyes, Methyl Orange (MO), Evans Blue (EB) and Remazol Brilliant Blue (RBB), were assayed as substrates for the HTS of laccase libraries. The three dyes were selected among a set of different dyes on the basis of their chemical structure since azoic (MO and EB) and anthraquinoid (RBB) dyes are the most common chromophores utilized in the dying industry [39]. Besides, they were directly oxidized by commercial HRPL (TvL). Changes in the absorption visible spectra carried out during the enzymatic oxidation of the three dyes provided the following λ_max_ for measuring their decolorization: 470, 605 and 640 nm for MO, EB and RBB, respectively. Figure 8 illustrates the oxidation of EB as an example. The elevated initial absorbance values of high dye concentrations were beyond the plate-reader's detection limit (Figure 8B), thus precluding the calculation of maximum velocities during decolorization of the three dyes by TvL (Figure 8C). However, we fixed 200 μM RBB and 50 μM MO and EB for the HTS assays because these concentrations provided perceptible responses (see Figure 8A, inset, for the *de visu* responses given by three replicates of the same laccase sample with each dye) and quantifiable decolorization rates (Figure 8D illustrates the linear response obtained with 2–100 mU laccase and 50 μM EB). Decolorization percentages of 49% for EB, 24% of RBB and 10% of MO were obtained after 3 h of reaction with TvL (10 mU). The presence of two hydroxyl substituent groups probably favors the rapid oxidation of Evans Blue (diazo) by laccase as compared to Methyl Orange (azo), whose redox potential is around +1 V (E_0_ = +0.961 V vs NHE [40]).

**Figure 9:**
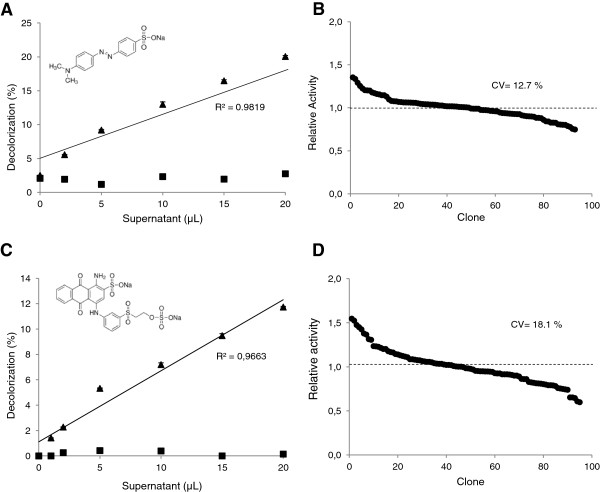
**Validation of HTS assays with Methyl Orange (A, B) and Remazol Brilliant Blue (C, D).** Linearity **(A, C)** and reproducibility **(B, D)** of decolorization assays with 50 μM Methyl Orange (20 h) and 200 μM Remazol Brilliant Blue (5 h), using crude extracts of *S. cerevisiae* microcultures expressing 3A4 HRPL (triangles) or R2 LRPL (squares). Reproducibility was tested with replicates of the same clone expressing 3A4 HRPL. The activities are plotted against the clones in descending order.

The oxidation of the three dyes was assayed in high-throughput format using crude extracts from *S. cerevisae* cells secreting the evolved HRPL 3A4 or the evolved LRPL R2. The latter was unable to oxidize any dye under the conditions used, whereas the HRPL decolorized the three dyes. The decolorizing yields obtained with 3A4 HRPL followed the same patterns as those obtained with TvL for the three dyes: EB > MO > RBB. Decolorization yields were around 39%, 11% and 6%, for EB, RBB and MO, respectively, after 6 h of reaction using 15 μl of supernatant from the well. The direct correlation between the volume of supernatant used and the decolorization values attained demonstrated the linearity of the three endpoint assays. Moreover, the CV values for the three dye-decolorizing assays were on average around 15%, which are acceptable to start directed evolution studies. The linearity and reproducibility of MO and RBB-based HTS assays are illustrated in Figure 9.

**Figure 10:**
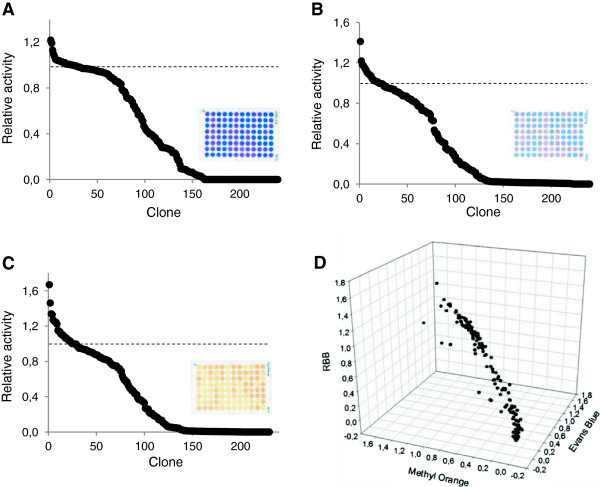
**Multi-screening with synthetic organic dyes.** Landscapes from the HTS of the mutagenic library from 3A4 HRPL using 50 μM Evans Blue **(A)**, 200 μM Remazol Brilliant Blue **(B)** and 50 μM Methyl Orange **(C)**. Laccase activities of the clones are depicted in descending order respecting the activity of the parent type. Direct correlation of the activities of the clones explored by the triple screening is shown in the 3D landscape **(D)**.

The three dye-decolorizing HTS assays were finally tested for screening a mutagenic library created by error prone PCR of the HRPL 3A4. Landscapes showed quantifiable differences among the decolorizing activities of the different clones and some slight laccase activity improvements respecting the parent type (Figure 10 A-C). In general, we observed a direct correlation among the activities of the different laccase mutants for the three dyes (Figure 10D).

**Figure 11:**
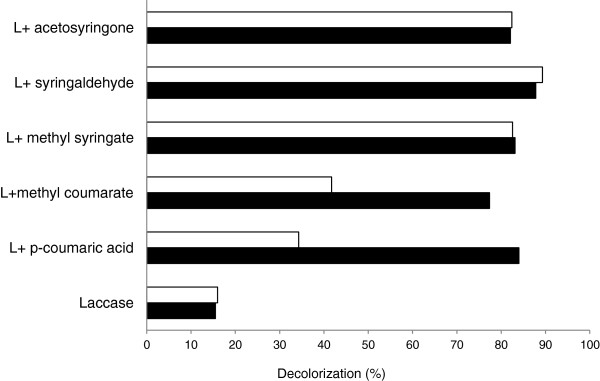
**Decolorization of 50 μM Methyl Orange by 10 mU of laccase from *M. thermophila *(white bars) or *T. villosa *(black bars), with or without phenolic mediators.** Mean values from two replicates after blank subtraction are shown (molar ratio of mediator/dye = 4; 5 h reaction time).

Textile wastewaters contain high concentrations of unfixed dyes (mostly azo and anthraquinone dyes) which cause great pollution problems due to their recalcitrance against conventional aerobic treatments and the generation of toxic aromatic intermediates during anaerobic treatment [39]. By contrast, oxidative decolorization of azo dyes by laccase produces a detoxifying effect [41,42]. These dye-decolorizing HTS methods can be useful for engineering laccases for decolorization and detoxification of synthetic organic dyes. In addition, the dye-decolorization assays can be used as indirect methods to evaluate the oxidative capability of laccase-mediator systems [43] or, more in particular, for screening laccase activity on natural mediators whose oxidation by laccase does not render colored products. This is the case of H-type phenolic compounds such as *p*-coumaric acid or methyl coumarate [6,27]. We tested the decolorization of MO by HRPL (TvL) and LRPL (MtL) in the absence or presence of phenolic mediators related to H or S-lignin units (Figure 11). In general, decolorization was significantly improved in the presence of S-type and H-type phenolic compounds, demonstrating their capability to mediate the enzymatic oxidation of the dye. Both laccases rendered similar decolorization values with the S-type mediators, whereas the decolorization attained in the presence of H-type mediators were much lower with MtL than with TvL. The low redox potential of S-type phenolic compounds (sinapic acid E_0_ = +590 mV, [44]; syringaldehyde E_0_ = + 660 mV, [45]) facilitates their oxidation by both enzymes. By contrast, the oxidation of methyl-coumarate and *p*-coumaric acid (E_0_ ≈ +700 mV,[46]), although thermodinamically feasible, is limited for the LRPL MtL (E_0_ at T1 copper site ≈ +470 mV), but not for the HRPL TvL (E_0_ ≈ +780 mV) [47], thus explaining the better decolorization values attained with the latter when using H-mediators. Nonetheless, the decolorization assay might still be useful as an indirect method for the *in vitro* evolution of fungal laccases towards H-type mediators whose oxidation cannot be detected by the naked-eye.

## Conclusions

We have devised and validated a set of colorimetric activity assays in high-throughput format for exploring laccase activity in mutant libraries generated by directed evolution. The assays are based on the enzymatic oxidation of natural redox mediators derived from lignocellulose and synthetic organic dyes. Besides, the use of violuric acid assay as reporter of laccase redox potential can be useful to preserve this significant property whilst evolving towards new functions. As we demonstrate here, these new colorimetric HTS assays are reproducible and reliable enough for contributing to face up to new evolution challenges. The engineering of laccase variants with better catalytic efficiencies towards key natural phenolic compounds, under preferred conditions, might be of relevance for the application of these enzymes in industrial processes of conversion of plant biomass. The dye-decolorizing HTS assays can be used for engineering *ad hoc* laccases to be applied in detoxification of textile industrial wastewaters. In addition, they can be used as indirect HTS assays for searching for better oxidation activities on phenolic mediators of interest, whose enzymatic oxidation cannot be detected in the visible spectrum.

## Methods

### Reagents and enzymes

Crude laccases from *Trametes villosa* (TvL) (NS- 51002) and *Myceliopthora thermophila* (MtL) (NS-51003) were purchased from Novozymes (Denmark). Reagents Methyl Orange (MO), Evans Blue (EB), Remazol Brilliant Blue (RBB), Sinapic acid, Acetosyringone (3′,5′-dimethoxy-4′-hydroxyacetophenone), Syringaldehyde (3,5-dimethoxy-4-hydroxybenzaldehyde), violuric acid (5-(Hydroxyimino)-2,4,6(1H,3H,5H)-pyrimidinetrione) and ABTS were purchased from Sigma–Aldrich.

### Culture media

Minimal medium contained 100 ml 67 g/l sterile yeast nitrogen base, 100 ml 19.2 g/l sterile yeast synthetic dropout medium supplement without uracil, 100 ml sterile 20% raffinose, 700 ml sterile double-distilled H_2_O (ddH_2_O), and 1 ml 25 g/l chloramphenicol. Yeast extract-peptone (YP 1.55X) medium contained 10 g yeast extract, 20 g peptone, and ddH_2_O to 650 ml. Expression medium contained 720 ml YP 1.55X, 67 ml 1 M KH_2_PO_4_, pH 6.0, buffer, 110 ml 20% galactose, 2 mM CuSO_4_, 25 g/l ethanol, 1 ml 25 g/l chloramphenicol, and ddH_2_O to 1,000 ml. The yeast extract-peptone-dextrose (YPD) solution contained 10 g yeast extract, 20 g peptone, 100 ml 20% sterile glucose, 1 ml 25 g/l chloramphenicol, and ddH_2_O to 1,000 ml. Synthetic complete (SC) dropout plates contained 100 ml 67 g/l sterile yeast nitrogen base, 100 ml 19.2 g/l sterile yeast synthetic dropout medium supplement without uracil, 20 g bacto agar, 100 ml 20% sterile glucose, 1 ml 25 g/liter chloramphenicol, and ddH_2_O to 1,000 ml. The SC drop-out plates to test the screening assays in solid format contained 10 μM CuSO_4_, 2 g/l galactose instead of glucose and 200 μM syringaldehyde, acetosyringone or sinapic acid.

### Oxidation assays with commercial laccases

Standard assay with 3 mM ABTS was used for initial measurement of laccase activities by recording the increase of absorption with time at 418 nm (ϵ_ABTS_^•+^ = 36,000 M^-1^ cm^-1^). Changes in the UV-Vis spectra of S-type phenolic substrates, dyes and violuric acid during oxidation by TvL or MtL (20 mU) in 100 mM tartrate buffer pH 4.0, were recorded in the spectrophotometer (Shimadzu UV-1800) to determine the corresponding λ_max_ and concentrations to be used in the HTS assays. Then, oxidation of S-type phenolics was measured by the increase of absorbance at 370 nm for syringaldehyde (2 mM), 520 nm for acetosyringone (2 mM) and 512 nm for sinapic acid (250 μM); decolorization of dyes was measured by the decrease of absorbance at 470 nm for MO (50 μM), 605 nm for EB (50 μM) and 640 nm RBB (200 μM); and oxidation of violuric acid (20 mM) was measured by the increase in absorbance at 515 nm. All measurements were carried out in buffer sodium tartrate pH 4.0 (250 μL final volume) in a plate reader (SPECTRAMax Plus 384, Molecular Devices).

### Micro-cultures of *S. cerevisiae* cells expressing laccase mutants

Colonies from yeast transformed cells were picked and transferred to 96-well plates where they were cultured in 50 μl of minimum medium for two days. Then, 160 μl of expression medium [22] were added and the plates were incubated during another three days. Micro-fermentations (210 μl) were carried out at 30°C and 200 rpm in a humidity shaker.

To determine laccase activity in the wells, plates were centrifuged and aliquots of the supernatant were transferred to new plates with the help of a liquid handler (Quadra96, Tomtec, USA). Target substrates in tartrate buffer pH 4.0 were added to a final volume of 250 μL and endpoint absorbances at the corresponding λ_max_ were measured in the plate reader, except for ABTS, which was measured in kinetic mode.

### Validation of the HTS colorimetric assays

#### Linearity of the endpoint assay

To test the linearity and sensitiveness of the assays, wells were inoculated with yeast cells expressing the evolved laccases R2 or 3A4. Un-inoculated wells were used as negative control. Micro-fermentations were carried out as mentioned above and, after centrifugation, different volumes of supernatant (1–30 μL) were transferred to new plates. Next, target substrates were added in tartrate buffer to a final volume of 250 μl and laccase activities were measured in the plate reader as described above.

#### Reproducibility of the endpoint assay (CV)

Yeast transformed cells expressing the evolved HRPL 3A4 were cultured in each well of the same 96-well plate and incubated as above mentioned. Thereafter, 30 μL of the supernatants containing the secreted laccase were transferred to new plates and 220 μl of the different substrates in tartrate buffer pH 4.0 were added. The reactions were kept during 24 h and the oxidation products were measured at the corresponding λ_max_ using the plate reader in endpoint mode.

### Construction of the mutagenic libraries

A small mutagenic library was created by error-prone PCR of the chimeric HRPL 3A4 [24] to test the HTS-assays. Reaction mix contained 5 μl 10× Taq buffer, 3% DMSO, 1.5 mM MgCl_2_, 0.01 mM MnCl_2_, 0.3 mM dNTP mix, 90 nM of primers RMLN and RMLC, 4.6 ng parent plasmid DNA (pJRα3A4) and 2.5 units of Taq DNA polymerase in a final volume of 50 μl. PCR cycles were 95° for 2 min; 28 cycles of 94° for 0.45 min, 53° for 0.45 min, 74° for 3 min; and 74° for 10 min. Reaction products were loaded into 0.8% agarose gels and 1.9 kb bands were cut and purified. 400 ng of this purified product was used to transform yeast together with 100 ng of the pJRoC30 expression vector previously linearized with BamHI and NotI.

A larger laccase library created by error-prone PCR and *in vivo* shuffling of selected chimeric laccases [24], was used for testing the sinapic acid assay. The error-prone PCR reactions of five chimeric laccases were the same as described above. Then, the amplified products were purified and jointly transformed in the yeast, using 133 ng of each parental insert and 200 ng of the linearized plasmid.

### High-throughput screening of laccase libraries

The endpoint colorimetric assays were tested in the abovementioned mutagenic libraries. Two hundred colonies were picked from SC-dropout plates and individual clones were grown in wells of 96-well plates as described above. Column 6 from each plate was inoculated with parent type, while well H1 was not inoculated (blank). After centrifugation, 30 μl supernatants were transferred to replica plates where 220 μl of 2 mM acetosyringone, 2 mM syringaldehyde, 20 mM violuric acid, 50 μM MO, 50 μM EB or 200 μM RBB in tartrate buffer pH 4 were added. The plates were briefly stirred, and the absorption at the corresponding λ_max_ (see above) were measured. The plates were incubated at room temperature in darkness and laccase activities were measured by the increase (mediators) or decrease (dyes) of color. Relative activities were calculated from the difference in absorption over time normalized against the parental type in the corresponding plate. The colorimetric assay with sinapic acid as substrate (250 μM) was evaluated with two thousand colonies from a larger laccase library, following the same procedure.

## Abbreviations

HTS: High-throughput screening; H: *p*-hydroxyphenyl lignin units; S: Syringyl lignin units; HRPL: High-redox potential laccase; LRPL: Low-redox potential laccase; TvL: *Trametes villosa* laccase; MtL: *Myceliophthora thermophila* laccase; R2: Evolved LRPL from MtL; 3A4: Evolved HRPL from shuffling of PM1 and *P. cinnabarinus* laccases; MO: Methyl orange; EB: Evans blue; RBB: Remazol brilliant blue.

## Competing interests

The authors declare that they have no competing interests.

## Authors’ contributions

IP and XC have equally contributed to this study. IP constructed the mutagenic libraries, and contributed to the development and validation of the colorimetric assays in high-throughput format and to figures design. XC carried out the assays with commercial enzymes, developed the dye-decolorizing assays in high-throughput format and performed the screening of mutant libraries. AIV developed the screenings assaysin solid format. MA made critical revision of the manuscript. SC coordinated the conception and design of the study and wrote the manuscript, which was read and approved by all the authors.
